# Sex and dependence related neuroanatomical differences in regular cannabis users: findings from the ENIGMA Addiction Working Group

**DOI:** 10.1038/s41398-021-01382-y

**Published:** 2021-05-06

**Authors:** Maria Gloria Rossetti, Scott Mackey, Praveetha Patalay, Nicholas B. Allen, Albert Batalla, Marcella Bellani, Yann Chye, Patricia Conrod, Janna Cousijn, Hugh Garavan, Anna E. Goudriaan, Robert Hester, Rocio Martin-Santos, Nadia Solowij, Chao Suo, Paul M. Thompson, Murat Yücel, Paolo Brambilla, Valentina Lorenzetti

**Affiliations:** 1 Department of Neurosciences, Biomedicine and Movement Sciences, Section of Psychiatry, University of Verona, Verona, Italy; 2 Department of Neurosciences and Mental Health, Fondazione IRCCS Ca’ Granda Ospedale Maggiore Policlinico, Milan, Italy; 3 Department of Psychiatry, University of Vermont, Burlington, VT USA; 4 Centre for Longitudinal Studies and MRC Unit for Lifelong Health and Ageing, IOE and Population Health Sciences, UCL, London, UK; 5 Department of Psychology, University of Oregon, Eugene, OR USA; 6 Department of Psychiatry, UMC Utrecht Brain Center, Utrecht University, Utrecht, The Netherlands; 7 BrainPark, Turner Institute for Brain and Mental Health, School of Psychological Sciences & Monash Biomedical Imaging Facility, Monash University, Melbourne, VIC Australia; 8 Department of Psychiatry, Université de Montreal, CHU Ste Justine Hospital, Montreal, QC Canada; 9 Department of Developmental Psychology, University of Amsterdam, Amsterdam, the Netherlands; 10 Department of Psychiatry, Amsterdam Institute for Addiction Research, University of Amsterdam, Amsterdam, Netherlands; 11 School of Psychological Sciences, University of Melbourne, Melbourne, VIC Australia; 12 Department of Psychiatry and Psychology, Hospital Clinic, IDIBAPS, CIBERSAM and Institute of Neuroscience, University of Barcelona, Barcelona, Spain; 13 School of Psychology and Illawarra Health and Medical Research Institute, University of Wollongong, Wollongong, NSW Australia; 14 Imaging Genetics Center, Mark and Mary Stevens Neuroimaging and Informatics Institute, Keck School of Medicine, University of Southern California, Marina del Rey, CA USA; 15 Department of Pathophysiology and Transplantation, University of Milan, Milan, Italy; 16 Neuroscience of Addiction & Mental Health Program, Healthy Brain and Mind Research Centre, School of Behavioural & Health Sciences, Faculty of Health Sciences, Australian Catholic University, Melbourne, VIC Australia

**Keywords:** Addiction, Neuroscience

## Abstract

Males and females show different patterns of cannabis use and related psychosocial outcomes. However, the neuroanatomical substrates underlying such differences are poorly understood. The aim of this study was to map sex differences in the neurobiology (as indexed by brain volumes) of dependent and recreational cannabis use. We compared the volume of a priori regions of interest (i.e., amygdala, hippocampus, nucleus accumbens, insula, orbitofrontal cortex (OFC), anterior cingulate cortex and cerebellum) between 129 regular cannabis users (of whom 70 were recreational users and 59 cannabis dependent) and 114 controls recruited from the ENIGMA Addiction Working Group, accounting for intracranial volume, age, IQ, and alcohol and tobacco use. Dependent cannabis users, particularly females, had (marginally significant) smaller volumes of the lateral OFC and cerebellar white matter than recreational users and controls. In dependent (but not recreational) cannabis users, there was a significant association between female sex and smaller volumes of the cerebellar white matter and OFC. Volume of the OFC was also predicted by monthly standard drinks. No significant effects emerged the other brain regions of interest. Our findings warrant future multimodal studies that examine if sex and cannabis dependence are specific key drivers of neurobiological alterations in cannabis users. This, in turn, could help to identify neural pathways specifically involved in vulnerable cannabis users (e.g., females with cannabis dependence) and inform individually tailored neurobiological targets for treatment.

## Introduction

Cannabis is the most widely used illicit substance on the planet and is the first drug of concern in treatment services nearly worldwide^1^. Sex differences are apparent in many aspects of cannabis use and dependence. For instance, males represent the majority of cannabis users^1,2^ and are more likely to become dependent^2^ but females progress more rapidly from recreational use to dependence and relapse more often^2–4^. Such differences have been partially attributed to sex-dependent underlying neurobiology^5,6^. For instance, the distribution and affinity of cannabinoid type 1 receptors (CB1Rs), which bind psychoactive compounds of cannabis (e.g., tetrahydrocannabinol (THC)), are affected by sex hormones and vary between males and females^5,6^. Thus, there may be sex differences in the neurobiological correlates of cannabis use.

Structural neuroimaging evidence in cannabis users shows mixed evidence for altered brain volumes in areas relevant to addiction-related cognitive processes (e.g., stress, learning, disinhibition)^7^ and that are high in CB1Rs^8^ (e.g., amygdala, hippocampus, prefrontal cortex (PFC), including the orbitofrontal cortex (OFC) and the anterior cingulate cortex (ACC), cerebellum and striatum^9–11^). A recent mega-analysis reported no significant volume differences between cannabis users and controls in these regions^12^. However, the literature to date^9,12^ has failed to account for putative moderators of volume alterations in cannabis users such as cannabis dependence status, which neuroscientific theories of addiction ascribe to profound neuroadaptations^13^, and confounders associated with cannabis use including tobacco and alcohol exposure.

The role of sex differences in volume alterations in cannabis users has also been under-investigated^14^. Emerging cannabis-by-sex effects were shown in the amygdala (i.e., female users > female controls)^15^, the PFC (i.e., female users > female controls and male users < male controls)^16^ and the OFC (i.e., female dependent users < female controls)^17^ but these were not replicated^18^ and were not found in other brain regions (e.g., cerebellum^19^, striatum^20^ parietal cortex^18^). Therefore, the differential effect of cannabis on the neuroanatomy of males and females remains elusive. Most published studies to date (i.e., 19 out of 30) have a male sampling bias, did not examine group-by-sex interactions^21^ or failed to account for drivers of neuroanatomical alterations (e.g., cannabis dependence, alcohol and tobacco use^11,12,22,23^).

Here we aimed to address these limitations by investigating brain volume differences associated with recreational and dependent cannabis use and their interaction with sex. We compared brain volumes in 129 regular cannabis users (of whom 59 were cannabis dependent) and 114 controls recruited from the ENIGMA Addiction Working Group while accounting for exposure to substances other than cannabis (i.e., alcohol and tobacco). We focused on a priori regions of interest (ROIs) that have been examined by at least three studies^9,24^ and showed volumetric differences (although not unanimously) between cannabis users and controls i.e., amygdala, hippocampus, nucleus accumbens (NAcc), insula, OFC, ACC and the cerebellum^20,24–28^. Also, the ROIs were selected for their high in CB1cannabinoid receptors based on autographic evidence CBR1s^8,29^ and for their key role in prominent neuroscientific theories of addiction^30,31^.

Based on previous structural MRI studies, we expected that (i) cannabis users (particularly dependent users) would show smaller volumes in some ROIs (i.e., amygdala, hippocampus, insula, OFC, ACC, cerebellar white matter)^24–26,32^ and larger volumes in other ROIs (i.e., NAcc, cerebellar grey matter)^20,27,28^ and (ii) there would be group-by-sex interactions within the OFC and the amygdala^15,17^.We also explored whether sex differences would emerge in other a-priori ROIs where these effects have not been examined (or found) so far^18–20^. Last, we explored separately in recreational and dependent users, if sex and substance use parameters (i.e., cannabis dosage, age of cannabis use onset, monthly standard drinks and monthly cigarettes) predicted brain volume of those ROIs that demonstrated significant group-by-sex interactions, after accounting for intracranial volume (ICV), age and IQ.

## Materials and methods

This study was pre-registered on the Open Science Framework (https://osf.io/spq2w).

MRI and behavioural data were obtained from seven research sites in accordance with the Declaration of Helsinki. All sites had obtained written informed consent from all participants. After primary data cleaning, three sites were excluded as they were missing information for monthly standard drinks and monthly cigarettes. Inclusion and exclusion criteria and key imaging, clinical and substance use assessment measures for the remaining four sites^27,33–35^ are shown in Supplementary Tables S1 and S2. Briefly, participants were excluded if they had psychiatric comorbidities; lifetime substance use (other than cannabis) greater than 5-to-100 times; MR contraindications or current use of psychotropic medications. We further excluded cannabis users who had abstained from cannabis for longer than 30 days (*n* = 15), and participants with significant MR image artefacts that undermined the validity of brain measures (*n* = 5) and missing IQ (*n* = 3), monthly standard drinks (*n* = 21) or monthly cigarettes (*n* = 3) data that were required as covariates for the analyses. The final sample included 243 participants, of whom 129 were regular cannabis users as defined at each site (38 females, mean age 27.54 ± 10.12), and 114 participants were non-cannabis using controls (33 females, mean age 26.19 ± 9.10).

### Measures

Participants’ demographic and substance use characteristics were assessed using semi-structured interviews at each site. These interviews assessed age, sex, IQ, monthly standard drinks, monthly cigarettes and cannabis use parameters (i.e., dosage, age at onset of use and dependence status). We standardised quantities across individuals by converting cannabis dosage (reported by participants in many forms shown in Supplementary Table S1) into standardised monthly ‘cones’ (defined here https://cannabissupport.com.au/media/1593/timeline-followback.pdf). Distributions of monthly standard drinks, cigarettes and cones were positively skewed, so were squared-root transformed prior to statistical analyses. Cannabis dependence status was available from three of the four sites and was used to segregate a three-site subsample (*n* = 206) into 59 dependent users (17 were females) with a mean age of 25 years, 49 recreational users (of which 20 were females) with a mean age of 27 years, and 98 non-cannabis using controls (including 33 females), with a mean age of 25 years. Cannabis dependence was determined using validated instruments with diagnostic cut-offs (i.e., > 3 for Mini Neuropsychiatry International Interview (MINI)^36^ and > 4 for the Severity of Dependence Scale (SDS)^37^).

### Structural MRI data acquisition and processing

Each site acquired structural T1-weighted MRI brain data which were prepared for analysis using FreeSurferv.5.3.0 (http://surfer.nmr.mgh.harvard.edu/), a fully automated MRI processing pipeline that identifies seven bilateral subcortical and 34 bilateral cortical ROIs^38,39^. Briefly, after automated Talairach transformation and removal of non-brain tissue and skull^40^ the T1-weighted images were used to segment brain tissues and to estimate the grey matter–white matter interface, which was used as the starting point for the 3D reconstruction of the cortical surfaces. Then, each subject’s cortical model was parcelled into ROIs according to the Desikan–Killiany atlas^39^ and surface-based cortical volumes were estimated at the ROI level for all participants. Following all automated processing and parcellation procedures, FreeSurfer was again utilized to extract absolute segmented volumes of subcortical regions. All FreeSurfer output underwent quality control at each site, according to ENIGMA standardized protocols (http://enigma.ini.usc.edu/protocols/imaging-protocols/), which included outlier detection and visual inspection of all data. Analyses were performed on a total of 10 bilateral ROIs i.e., hippocampus, amygdala, NAcc, insula, medial OFC, lateral OFC, rostral ACC, caudal ACC, cerebellum grey matter, and cerebellum white matter. Left and right hemispheres were considered separately for each ROI.

### Statistical analyses

Chi-squared tests assessed differences in sex distributions between groups (i.e., recreational cannabis users, dependent cannabis users, controls).

A series of mixed-effect models were run to examine group, sex and group-by-sex differences for demographic and substance use characteristics, and brain volumes. This technique statistically accommodates dependency between observations in a nested design (i.e., participants within sites)^41^. *Site* was treated as a random effect to account for the systematic site-level variation in the dependent variables expected to occur from differences in scanners, protocols and assessment tools.

#### ROI volumes in cannabis users and controls of the full sample (*n* = 243; 4 sites)

In the full sample, we examined the impact of factors including group (controls, cannabis users [encapsulating both recreational and dependent users]), sex (male, female) and group-by-sex, on ROI volumes as dependent variables, controlling for ICV, age, IQ, monthly standard drinks, and monthly cigarettes. Group-by-sex interaction effects with a nominal significance level of p(uncorrected) < 0.05 were interrogated using pairwise comparisons.

#### ROI volumes in dependent cannabis users, recreational users and controls of the subsample with data on cannabis dependence status (*n* = 206; 3 sites)

We replicated the analysis above in the three-site subsample where cannabis dependence status was available, using group (controls, dependent cannabis users, recreational cannabis users), sex (male, female) and group-by-sex as factors, ROI volumes as dependent variables, and ICV, age, IQ, monthly standard drinks and monthly cigarettes as confounding variables. We also controlled for monthly cannabis dosage (i.e., “cones”) as these were significantly higher in dependent cannabis users than recreational users. Group-by-sex interaction effects with a nominal significance level of p(uncorrected) < 0.05 were interrogated using pairwise comparisons.

#### Exploratory associations between ROI volumes and substance use levels in dependent and recreational cannabis users from the three-site subsample

Exploratory analyses were run separately in dependent (*n* = 59) and recreational cannabis users (*n* = 49) of the three-site subsample where information on cannabis dependence status was available and for ROIs that were significantly affected by group-by-sex interactions. Specifically, we examined if ROIs volume was predicted by sex and substance use levels (i.e., age at onset of cannabis use, monthly cannabis cones, monthly standard drinks and monthly cigarettes) controlling for age, IQ and ICV.

All volumetric results were corrected for multiple comparisons using a False Discovery Rate (FDR) corrected statistical threshold of p(FDR) < 0.05^42^. Effect sizes were estimated for the significant p(uncorrected) < 0.05 group and group-by-sex effects using Cohen’s *d* and based on the marginal means predicted by the model. All analyses were run with STATA 14 (StataCorp; 2015).

## Results

### Samples characteristics

Table 1 shows demographic and substance use characteristics and brain volumes of the original sample (4 sites). Cannabis users (*n* = 129) versus controls (*n* = 114) did not differ in sex distribution, age, IQ or monthly standard drinks, but smoked more monthly cigarettes. These variables were matched between recreational cannabis users (*n* = 49), dependent cannabis users (*n* = 59) and controls (*n* = 98) of the subsample with information on cannabis dependence status (three sites). However, dependent cannabis users compared to recreational users were, on average, older and smoked more cannabis cones per month (Supplementary Table S3).

**Table 1:** Demographic, substance use characteristics and brain volumes of cannabis users (*n* = 114) and controls (*n* = 129) from the full sample (Mean, SD).

		HC				CB				Group			Sex						Site^§^
		Males (*n* = 81)		Females (*n* = 33)		Males (*n* = 91)		Females (*n* = 38)		(CB vs HC)			(Males vs Females)			Group-by-Sex			
		Mean	SD	Mean	SD	Mean	SD	Mean	SD	*β*	95% CI	*p*	*β*	95% CI	*p*	*β*	95% CI	*p*	Var
Sample characteristics																			
Age, yrs		26.47	9.16	25.50	9.06	26.83	9.99	29.24	10.37	2.05	−1.49, 5.60	0.256	1.49	−1.79, 4.77	0.373	−2.13	−6.32, 2.06	0.319	0.44
IQ		109.31	12.00	106.21	11.70	101.43	12.43	101.84	10.41	−4.81	−10.12, 0.51	0.076	4.31	−0.56, 9.19	0.083	−3.14	−9.43, 3.16	0.329	0.10
Alcohol, StDr/mo		20.91	22.92	17.60	23.10	32.69	36.57	18.74	19.27	0.24	−12.72, 13.19	0.971	7.23	−4.53, 18.99	0.228	11.03	−4.32, 26.38	0.159	0.04
Tobacco, Cig/mo		25.95	95.83	51.09	110.03	262.10	243.89	265.57	238.28	209.08	122.72, 295.45	<0.001***	−23.08	−101.65, 55.48	0.565	26.92	−75.37, 129.22	0.606	0.50
Cannabis																			
Onset of use, yrs		–	–	–	–	15.58	3.00	15.06	2.20	–	–	–	1.30	−0.56, 3.16	0.170	–	–	–	0.16
Dosage, cones/mo		–	–	–	–	349.22	354.86	263.84	221.74	–	–	–	−5.04	0.918	0.918	–	–	–	0.11
Dependence, N		–	–	–	–	42	17	–	–	–	–	–	–	–	–	–	–	–	
ICV, 10^^6^		1.62	0.16	1.40	0.17	1.58	0.14	1.41	0.18	− 0.02	−0.08, 0.04	0.564	0.200	0.14, 0.26	<0.001***	−0.04	−0.11, 0.04	0.355	0.24
Brain volumes (mm^3^)																			
Amygdala	L	1763.01	292.20	1678.16	291.87	1679.82	246.64	1575.18	244.72	−27.12	−122.59, 68.35	0.578	94.14	5.01, 183.26	0.038	6.16	5.01, 183.26	0.909	0.52
	R	1877.83	288.21	1664.50	240.89	1764.58	274.17	1593.80	203.18	38.13	−54.93, 131.18	0.422	161.32	74.45, 248.19	<0.001***	−47.13	−149.92, 55.66	0.369	0.51
Hippocampus	L	4528.68	515.90	4271.71	358.52	4348.256	455.43	4139.88	500.01	−15.61	−230.64, 199.43	0.887	71.99	−123.68, 267.67	0.471	32.44	−205.35, 270.23	0.789	0.03
	R	4673.32	470.76	4377.24	383.79	4438.95	443.65	4286.33	405.05	57.44	−136.39, 251.27	0.561	146.10	−31.76, 323.95	0.107	−78.05	−292.33, 136.23	0.475	0.05
NAcc	L	664.90	193.93	601.42	195.24	629.18	186.42	544.63	201.53	18.56	−35.08, 72.20	0.498	34.07	−16.02, 84.18	0.182	−6.198	−65.45, 53.06	0.838	0.63
	R	685.99	164.37	621.58	186.611	644.23	167.47	573.18	169.31	23.73	−21.18, 68.65	0.300	30.76	−11.19, 72.71	0.151	−19.84	−69.44, 29.77	0.433	0.68
Insula	L	7215.20	855.05	6584.58	917.97	6890.40	700.23	6448.53	876.73	−93.13	−387.16, 200.90	0.535	138.92	−134.41, 412.25	0.319	−14.21	−339.08, 310.66	0.932	0.19
	R	7273.36	808.25	6731.00	611.93	7071.40	818.54	6470.47	772.18	−214.32	−515.91, 87.27	0.164	−39.69	−318.31, 238.93	0.780	238.43	−94.89, 571.74	0.161	0.09
OFC																			
*lateral*	L	8343.56	946.82	7793.00	748.39	8176.92	982.49	7523.92	713.03	−90.77	−447.65, 266.11	0.618	199.90	−132.48, 532.27	0.239	169.87	−224.40, 564.15	0.398	0.27
	R	8078.38	971.27	7375.91	1141.61	7744.87	1052.74	7039.63	828.42	−404.94	−773.76, −36.11	0.031*^a^	70.76	−272.63, 414.14	0.686	204.72	−202.76, 612.21	0.325	0.25
*medial*	L	5410.72	688.31	4855.97	504.75	5315.19	705.37	4736.89	666.16	−52.19	−315.19, 210.82	0.697	11.52	−232.58, 255.62	0.236	175.89	−114.72, 466.51	0.236	0.15
	R	5678.17	677.78	5386.58	544.92	5466.51	725.39	5041.79	450.31	−269.28	−536.11, 2.46	0.048*^b^	56.47	−191.89, 304.83	0.656	176.20	−118.60, 470.99	0.241	0.23
ACC																			
*rostral*	L	3060.65	612.19	2881.24	560.99	2862.80	614.80	2783.53	401.59	−96.06	−339.20, 147.07	0.439	5.38	−220.66, 231.43	0.963	−45.47	−314.107, 223.17	0.740	0.19
	R	2386.26	439.26	2267.18	497.40	2228.21	497.27	2143.76	340.83	−82.13	−293.81, −129.56	0.447	33.76	−162.12, 229.64	0.736	−15.31	−249.24, 218.62	0.898	0.10
*caudal*	L	2073.33	490.51	2066.30	435.04	1983.84	545.03	1875.63	441.37	−171.99	−402.32, 58.35	0.143	−252.14	−453.11, −51.16	0.014*	152.34	−102.72, 407.40	0.242	0.00
	R	2400.81	560.96	2268.97	681.49	2282.64	553.75	2229.16	439.47	−12.56	−272.12, 247.01	0.094	169.05	−171.72, 409.83	0.169	−48.32	−335.13, 2838.51	0.741	0.14
Cerebellum																			
*GM*	L	56930.18	7414.39	52211.16	7261.82	57421.36	6548.82	52735.63	6271.57	153.45	−2244.21, 2551.11	0.900	3311.98	1075.90, 5548.07	0.004**	1411.51	−1237.25, 4060.28	0.296	0.37
	R	57852.19	7989.12	53564.62	8008.75	58227.42	7211.22	54157.91	8226.83	−84.54	−2692.11, 2523.04	0.949	3007.19	574.50, 5439.89	0.015*	1323.84	−1556.78, 4204.46	0.368	0.41
*WM*	L	15451.41	2921.70	14864.99	2227.40	15124.64	2336.08	14267.18	1797.71	−577.66	−1458.06, 302.73	0.198	−297.85	−1119.06, 523.36	0.447	816.76	−155.83, 1789.36	0.100	0.39
	R	15831.57	3332.71	15093.30	1975.81	15612.82	2817.39	14374.62	1773.20	−584.08	−1575.38, 407.21	0.248	−240.14	−1165.07, 684.79	0.611	1119.24	24.15, 2214.33	0.045*^c^	0.43

*ACC* anterior cingulate cortex, *β* beta, *CB* cannabis users, *CI* confidence interval, cones = standardized measure of cannabis dosage, see https://cannabissupport.com.au/media/1593/timeline-followback.pdf, *Cig* cigarettes, *GM* grey matter, *ICV* intracranial volume, *HC* controls, *L* left, *mo* monthly, *OFC* orbitofrontal cortex, *R* right, *StDr* standard drinks, *Var* variation, *WM* white matter, *yrs* years. ^§^Site level variation based on intraclass correlation (ICC). Differences between groups for sex distribution were measured with chi^2^ test (*χ*^*2*^ = 0.01, *p* = 0.93).

*p(unc) < 0.05, **p(unc) < 0.01, ***p(unc) < 0.001.

^a^*d* = −0.11.

^b^d = −0.08.

^c^Female CB < male CB (*β* = −879.10, *p* = 0.045, *d* = −0.12).

### Volumetric findings

#### Regular cannabis users versus controls from the full sample (4 sites)

In the full sample, regular cannabis users (including both dependent and recreational users) compared to controls had smaller volumes in both the right medial OFC (*β* = −269.28*, p(uncorrected) =* 0.048) and in the right lateral OFC (*β* = −404.94*, p(uncorrected)* *=* 0.031). Also, a group-by-sex interaction was observed in the right cerebellar white matter (*β* = 1119.24*, p(uncorrected)* *=* 0.045). Post-hoc pairwise comparisons showed that female cannabis users had, on average, smaller volumes than male cannabis users. These group and group-by-sex interaction effects had a small effect size and did not survive FDR correction (Table 1).

#### Dependent cannabis users versus recreational users and controls from the three-site subsample

**Fig. 1:**
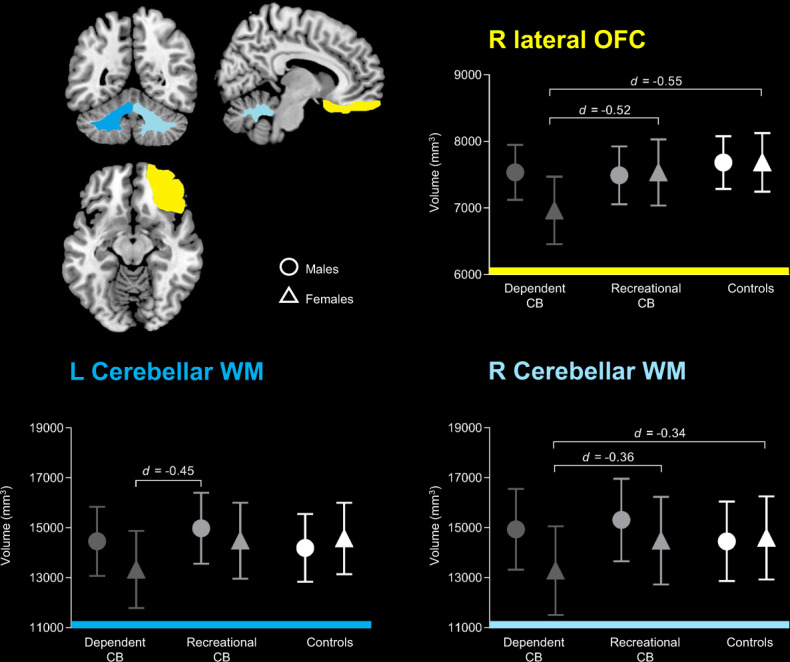
Predicted cerebellar white matter (WM) and right lateral orbitofrontal cortex (OFC) volumes in cannabis users (CB) with and without dependence and controls. Vertical and horizontal bars represent 95% confidence interval and group-by-sex effects, respectively.

Volumetric findings from the three-site subsample are shown in Table 2 and Fig. 1. Dependent cannabis users had smaller volumes of the right cerebellar white matter and right lateral OFC compared to both recreational users (*β* = −1269.72, *p(uncorrected)* *=* 0.42 and *β* = −564.93, *p(uncorrected)* *=* 0.025) and controls (*β* = −1564.76, *p(uncorrected)* *=* 0.24 and *β* = −702.11*, p(uncorrected)* *=* 0.012).

**Table 2:** Group, sex and group-by-sex effects in recreational cannabis users (*n* = 49), dependent cannabis users (*n* = 59) and controls (*n* = 98) from the three-site subsample with data for cannabis dependence status.

		Group									Sex						Site^§^
		(dependent CB vs recreational CB)			(dependent CB vs HC)			(recreational CB vs HC)			(Males vs Females)			Group-by-Sex			
		*β*	(95% CI)	*p*	*β*	(95% CI)	*p*	*β*	(95% CI)	*p*	*β*	95% CI	*p*	*β* (95% CI)	(95% CI)	*p*	Var
Brain volumes (mm^3^)																	
Amygdala	L	16.37	−115.85, 148.70	0.808	−197.83	−244.61, 48.95	0.191	−114.20	−245.18, 16.77	0.087	99.66	4.72, 194.60	0.040*	−38.36	−174.89, 98.16	0.582	0.58
	R	−25.05	−150.76, 100.66	0.696	13.76	−125.67, 153.20	0.847	38.82	−85.61, 163.24	0.541	172.80	83.40, 262.20	<001***	−58.37	−188.06, 71.32	0.378	0.58
Hippocampus	L	453.77	−221.38, 328.91	0.702	−31.70	−337.34, 273.94	0.839	−85.47	−356.62, 185.69	0.537	53.82	−135.67, 243.30	0.578	−11.52	−295.88, 272.86	0.937	0.03
	R	−133.11	−386.45, 120.22	0.303	−63.80	−345.06, 217.47	0.657	69.15	−180.80, 319.43	0.587	118.58	−58.27, 295.44	0.189	0.15	−261.81, 261.49	0.999	0.06
NAcc	L	−74.85	−149.74, 0.04	0.051	−41.69	−124.76, 41.37	0.325	33.15	−40.97, 107.29	0.236	35.57	−17.88, 89.03	0.192	13.75	−63.51, 91.01	0.727	0.65
	R	−51.93	−113.33, 9.47	0.097	−18.71	−86.83, 49.39	0.590	33.21	−27.58, −94.00	0.284	21.85	−21.95, 65.65	0.328	−14.87	−78.22, 48.47	0.380	0.88
Insula	L	−290.49	−688.94, 107.96	0.153	−169.37	−611.45, 272.70	0.453	121.12	−273.01, 515.24	0.547	90.08	−192.79, 372.96	0.533	172.93	−238.28, 584.13	0.410	0.21
	R	−204.12	−60.63, 195.39	0.317	−313.38	−756.88, 130.13	0.166	−109.25	−341.65, 218.83	0.587	−64.29	−343.62, 215.03	0.652	318.70	−93.88, 731.29	0.130	0.07
OFC																	
*lateral*	L	−429.13	−900.90, −42.62	0.075	−201.77	−725.12, 321.57	0.450	227.36	−725.12, 321.57	0.340	173.94	−161.72, 509.60	0.310	462.21	−24.58, 949.01	0.063	0.32
	R	−564.93	−1058.51, −71.35	0.025*^a^	−702.11	−1249.80, −153.41	0.012*^b^	−137.17	−625.22, 350.87	0.582	−3.27	−351.64, 345.11	0.985	575.33	65.87, 1084.78	0.027*^c^	0.15
*medial*	L	−201.33	−549.49, 146.84	0.257	−182.06	−568.43, 204.31	0.356	19.26	−324.91, 363.44	0.913	23.27	−222.03, 268.57	0.853	127.42	−231.99, 486.82	0.487	0.13
	R	−215.47	−561.45, 130.51	0.222	−278.42	−662.29, −105.44	0.155	−62.95	−405.18, 279.28	0.496	76.62	−171.78, 319.02	0.557	184.60	−172.46, 541.65	0.311	0.21
ACC																	
*rostral*	L	−233.80	−543.58, 75.98	0.139	−203.63	−492.21, 84.95	0.167	30.16	−247.86, 308.19	0.832	4.84	−218.22, 227.89	0.966	−78.23	−403.14, 246.68	0.637	0.25
	R	−89.39	−371.23, 192.45	0.547	−126.39	−389.15, 136.36	0.346	−37.01	−290.17, 216.15	0.774	47.72	−153.80, 249.25	0.643	−43.38	−339.29, 252.53	0.774	0.10
*caudal*	L	−208.86	−508.63, 90.91	0.172	−274.13	−555.09, 6.83	0.056	−65.27	−336.19, 205.65	0.637	−265.70	−469.06, −62.357	0.010*	224.09	−93.02, 541.19	0.166	0.00
	R	−5.77	−343.00, 331.46	0.973	−55.018	−369.24, 259.21	0.731	−49.25	−351.98, 253.49	0.750	172.43	−69.87, 414.73	0.163	−158.59	−512.40, 195.23	0.380	0.18
*medial*	L	−201.33	−549.49, 146.84	0.257	−182.06	−568.43, 204.31	0.356	19.26	−324.91, 363.44	0.913	23.27	−222.03, 268.57	0.853	127.42	−231.99, 486.82	0.487	0.13
	R	−215.47	−561.45, 130.51	0.222	−278.42	−662.29, −105.44	0.155	−62.95	−405.18, 279.28	0.496	73.62	−171.78, 319.02	0.557	184.60	−172.46, 541.65	0.311	0.21
Cerebellum																	
*GM*	L	−738.06	−3934.90, 2458.37	0.651	−2.26	−3534.43, 3547.96	0.999	740.32	−2423.29, 3903.93	0.646	3461.28	1189.26, 5733.30	0.003**	1676.65	−1621.32, 4974.63	0.319	0.47
	R	−1216.89	−4765.03, 2331.20	0.501	−261.34	−3674.48, 4197.17	0.896	1478.23	−2033.54, 4990.01	0.409	3110.34	584.19, 5636.49	0.016*	2009.77	−1651.08, 5670.61	0.282	0.48
*WM*	L	−1141.32	−2285.33, −2.70	0.051	−1193.07	−2462.19, 76.06	0.065	−51.74	−1183.74, 1080.25	0.929	−377.17	−1188.80 434.47	0.362	1507.86	327.38, 2688.33	0.012*^d^	0.32
	R	−1269.72	−2495.49, −43.93	0.042*^e^	−1564.76	−2924.55, −204.97	0.024*^f^	−295.05	−1508.04, 917.95	0.634	−136.99	−1007.92, 733.94	0.758	1786.54	521.74, 3051.34	0.006**^g^	0.36

*ACC* anterior cingulate cortex; *β* beta, *CB* cannabis users, *CI* confidence interval, *GM* grey matter, *L* left, *HC* controls, *OFC* orbitofrontal cortex, *R* right, *Var* variation, *WM* white matter. ^§^Site level variation based on intraclass correlation (ICC).

*p(unc) <0.05, **p(unc) <0.01, ***p(unc) <0.001.

^a^*d* = −0.10.

^b^*d* = −0.17.

^c^Female dependent CB < female recreational CB (*β* = −564.93, *p* = .025, d = −0.52), female HC (*β* = −702.11, *p* = .012, *d* = −0.55), male dependent CB (*β* = *−574.76, p* = .010, *d* = *−0.41*), male recreational CB (*β* = *−527.65, p* = .022, *d* = −0.45) and male HC (*β* = *701.54, p* = .010, *d* = −0.43).

^d^Female dependent CB < male dependent CB (*β* = −1136.554*, p* = .028, *d* = −0.26) and male recreational CB (*β* = −1652.85*, p* = .002, *d* = −0.45);

^e^*d* = −12.

^f^*d* = −0.09;

^g^Female dependent CB < female recreational CB (*β* = −1269.72, *p* = 0.042, *d* = −0.36), female HC (*β* = −1564.76, *p* = 0.024, *d* = −0.34), male dependent CB (*β* = −1620.06*, p* = 0.004, *d* = −0.32), male recreational CB (*β* = −2029.51*, p* < 0.001, *d* = −0.48) and male HC (*β* = −1398.28*, p* = 0.039, *d* = −0.23).

There was also a significant group-by-sex effect on the volumes of the cerebellar white matter (left: *β* = 1507.86, *p(uncorrected)= 0.12;* right: *β* = 1786.54*, p(uncorrected)* *=* 0.006*)* and the right lateral OFC (*β* = 575.33*, p(uncorrected)* *=* 0.027). Particularly, pairwise analyses demonstrated (i) smaller left cerebellar white matter in female dependent cannabis users compared to male recreational users and male dependent users and (ii) smaller right cerebellar white matter and right lateral OFC volumes in female dependent cannabis users relative to recreational users and controls of both sexes.

#### Association between cannabis, alcohol and tobacco use levels and ROI volumes separately in cannabis users with and without dependence from the three-site subsample

As shown in Table 3, smaller cerebellar white matter volumes in dependent cannabis users were significantly predicted by female sex (i.e., male = 1; female = 0) in both left (*β* = 1128.06*, p(FDR)* = 0.023) and right (*β* = 1352.56, *p(FDR)* = 0.011) hemisphere while smaller right lateral OFC volumes were significantly predicted by both female sex (*β* = 505.68*, p(FDR)* = 0.028) and more monthly standard drinks (*β* = *−*111.54*, p(FDR)* = 0.003). In recreational cannabis users, more monthly standard drinks predicted smaller right cerebellar white matter volumes (*β* = −254.22*, p(uncorrected)=* 0.033), but this effect did not survive FDR correction. No other predictor was significantly associated with cerebellum white matter and OFC volumes.

**Table 3:** Associations between regional brain volumes and substance use levels separately in cannabis users with and without dependence (three-site subsample).

			Dependent CB (*n* = 59)			Recreational CB (*n* = 49)		
			*β*	(95% CI)	*p*	*β*	(95% CI)	*p*
**Brain volumes (mm**^**3**^**)**								
OFC								
*lateral*	R	Sex^a^	505.68	53.45, 957.91	0.028*	−197.09	−770.26, 376.07	0.500
		Cannabis use onset, yrs	73.57	−28.24, 175.37	0.157	4.65	108.99, 118.29	0.936
		Cannabis dosage, cones/mo	−0.76	−32.92, 31.40	0.963	12.45	−24.65, 49.56	0.511
		Cigarettes/mo	9.02	−13.28, 31.33	0.428	6.21	−25.84, 38.27	0.704
		Standard drinks/mo	−111.54	−185.14, −37.94	0.003**	−42.03	−122.10, 38.04	0.304
Cerebellum								
WM	L	Sex^a^	1128.06	154.65, 2101.48	0.023*	578.83	−906.20, 2063.86	0.445
		Cannabis use onset, years	−20.14	−230.05, 189.77	0.851	88.65	−204.05, 381.36	0.553
		Cannabis dosage, cones/mo	−16.06	−85.10, 52.99	0.649	−8.73	−102.90, 85.44	0.856
		Cigarettes/mo	22.23	−23.79, 68.25	0.344	5.56	−76.04, 87.16	0.894
		Standard drinks/mo	−14.08	−138.04, 166.21	0.856	−118.14	−321.66, 85.37	0.255
	R	Sex^a^	1352.56	303.86, 2401.25	0.011*	1396.28	−303.28, 3095.85	0.107
		Cannabis use onset, years	9.39	−215.40, 234.19	0.935	61.40	−274.05, 396.84	0.720
		Cannabis dosage, cones/mo	9.90	−64.49, 84.28	0.794	36.48	−71.84, 144.81	0.509
		Cigarettes/mo	4.83	−44.46, 54.11	0.848	3.12	−90.66, 96.89	0.948
		Standard drinks/mo	40.24	−122.73, 203.20	0.628	−254.22	−488.24, −20.20	0.033*

Note: only ROIs that demonstrated significant group-by-sex effects were included in the analysis.

*CB* cannabis users, *CI* confidence interval, *L* left, *OFC* orbitofrontal cortex, *R* right, *WM* white matter.

^a^Male =1; Female = 0.

*p(unc) < 0.05, **p(unc) < 0.01, *p(FDR)* < 0.05.

## Discussion

### Summary of the results

The results of this multi-site MRI study partially confirmed our hypotheses. Specifically, group and group-by-sex effects emerged in the lateral OFC and the cerebellar white matter of cannabis users versus controls. These effects had small-to-moderate effect sizes and did not survive FDR correction. Yet, cannabis users versus controls did not show volumetric differences in the amygdala, hippocampus, insula, ACC, NAcc and cerebellar grey matter. Last, in recreational and dependent cannabis users, we found that lateral OFC and cerebellar white matter volumes were predicted by sex and alcohol dosage, but not cannabis use measures.

### Sex and cannabis dependence related differences between regular cannabis users and controls

We showed that in cannabis users, being female and dependent on cannabis was associated with smaller right lateral OFC and cerebellar white matter volumes.

Our report of smaller cerebellar white matter and OFC volumes in cannabis users versus controls is consistent with previous reports^9,10^ and with neuroscientific theories of addiction that implicate these regions in dependent, habitual substance use and related increased salience to substance-related stimuli, disinhibition, stress and craving^13,43,44^.

However, to date it remains to be clarified whether such alterations are the results of neuroadaptations associated with the development of addiction^45^ or due to neurotoxicity related to chronic exposure to cannabis^46^.

A key novel finding is that there was a significant interaction between female sex and cannabis dependence on cerebellar white matter and lateral OFC volumes suggesting that sex may moderate brain volume differences associated with cannabis dependence. To our knowledge, we are the first to report a group-by-sex effect in the cerebellar white matter of people with cannabis dependence. Our findings are in line with those from previous studies where sex differences were not examined^22,26^ and corroborate recent models of addiction that have reconsidered the cerebellum as a key region that play a modulating role between motor, reward, motivation and cognitive control systems via its functional connections with the corticostriatal-limbuic circuitry^31,43^.

Interestingly, we found that, within the group of dependent users, being female predicted volume reductions of those regions (i.e., lateral OFC, cerebellar white matter), over and above the effect of cannabis dosage, age of cannabis use onset and monthly standard drinks and monthly cigarette use. Thus, female sex may represent a vulnerability factor to develop volumetric alterations in this group.

The mechanisms behind smaller volumes of the cerebellum and OFC in female dependent cannabis users remain to be clarified. Pre-existing neurostructural sex differences in these regions have been shown in normative samples (e.g., smaller volume in males versus females^47,48^) and might represent a neurobiological vulnerability predating cannabis use^49,50^. Yet, sex differences have been reported within the endocannabinoid system^5,51^. For example, chronic cannabinoid exposure in rats leads to a more marked downregulation and desensitization of CB1Rs within the cerebellum and the OFC^51,52^. Notably, CB1Rs receptors are widely expressed on both neurons and glial cells (e.g., oligodendrocytes and oligodendroglial cells)^53^^,^^54^. Thus, the downregulation of cannabinoid receptors with long-term cannabis exposure might also suppress glial cells function^54^^,^^55^ and thereby alter white matter structures^26^. Interestingly, emerging evidence shows that microglial activation underlies cerebellar deficits produced by repeated cannabis exposure^56^. Sex differences in the endocannabinoid system have been ascribed to females experiencing stronger cannabis craving, withdrawal symptoms^2,57^, and psychoactive effects of the cannabinoid THC that confers addiction liability^17,58^, and may contribute to a faster escalation from regular use to dependence noted in female cannabis users^2–4^. Interestingly, preliminary evidence in a separate study of young adults showed that smaller OFC volume predicted cannabis use, suggesting that structural abnormalities in the OFC might contribute to risk for cannabis exposure^59^. Moreover, a significant association emerged between cerebellar white matter integrity and self-reported craving in people at risk of cannabis use disorders ^60^.

Longitudinal neuroimaging studies are required to extend these findings while accounting for sex differences predating/following cannabis dependence and in the transition from recreational to dependent cannabis use. Even so, the cross-sectional nature of this study did not allow us to determine causality versus pre-existing sex-related brain differences that may predict future dependent versus recreational cannabis use. Further longitudinal studies are needed to disentangle this issue.

### Associations between ROI volumes and alcohol standard drinks in recreational and dependent cannabis users

Of note, in dependent cannabis users, smaller OFC volumes were also associated with monthly standard drinks which is in line with evidence from structural MRI studies in alcohol users^12,61,62^. As such, one could speculate that alcohol use may have driven OFC reductions in dependent cannabis users compared to recreational users and controls. However, all between-group analyses accounted for monthly standard drinks and other important covariates (i.e., ICV, IQ, age, IQ, monthly cigarettes). Yet, cannabis users (with and without dependence) and controls were matched by the number of monthly standard drinks (Table 1 and Table S3). Alternatively, it may be possible that cannabis dependent users are more vulnerable to alcohol exposure than non-dependent users. Future studies comparing recreational and dependent users with and without alcohol co-use may help disentangle this issue.

Similarly, we found a marginally significant association between monthly standard drinks and smaller cerebellar white matter volumes in recreational cannabis users. This is in line with previous evidence from structural MRI studies showing cerebellar white matter changes in alcohol users ^43^ and underlines the need to systematically account for entrenched alcohol exposure in cannabis using samples.

### Negative findings

We did not find volumetric alterations within distinct ROIs in cannabis users compared to controls, specifically in the amygdala, hippocampus, insula, NAcc and ACC. This is partially in line with prior work that found both presence^19,20,24,25,32^ and absence^20,63^ of alterations of these ROIs in cannabis users compared to controls. Our work extends previous negative findings in recreational and dependent cannabis users within both sexes, after controlling for several key confounders (ICV, age, IQ, alcohol and tobacco use). The inconsistently reported volumetric differences in (recreational and dependent) cannabis users suggest that neuroanatomical alterations of ROIs that are implicated in neuroscientific theories of addiction^13,30^, may not be a core feature of cannabis use neurobiology.

Moreover, in contrast with prior work^9,24,64^, ROI volumes in our sample of cannabis users were not predicted by age at cannabis use onset or by cannabis dosage (i.e., monthly cannabis cones). One difference between our study and prior reports (in which specific variables significantly predict brain volumes) is that most prior studies, unlike this study, did not account for multiple relevant variables including sex, cannabis dependence, IQ and alcohol and tobacco use. Our findings emphasize a need to rethink the role of patterns of cannabis use versus dependence as well as variables associated with cannabis exposure^65,66^, as drivers of neuroanatomical differences between cannabis users and controls.

### Limitations

Our findings should be considered with caution. First, the size of the reported effects was small-to-medium and suggests that only a sub-set of cannabis users show smaller volumes (e.g., those with a longer history of cannabis use or greater severity of cannabis dependence). Replication studies in larger samples are required to identify the characteristics that confer vulnerability to develop brain alterations. Second, the inter-study variability in MRI (e.g., MR scanner magnetic field strength, manufacturer, acquisition parameters) and behavioural testing protocols may have confounded our study results. We mitigated this issue by using standardized high-quality MR quality check protocols^67,68^ and a multi-level statistical approach that accounts for error due to systematic differences between distinct study samples. Similarly, our findings on group and group-by-sex differences may have been confounded by the fact that number of monthly cigarettes was greater in cannabis users compared to controls. However, we controlled for differences in monthly cigarettes in all analyses. As such, we are confident that we accounted for the impact of this variable in estimating the result. Yet, future studies in groups carefully matched on tobacco use are needed to unpack the concurrent impact of cannabis and tobacco use on the brain of cannabis using samples with entrenched tobacco use. Third, our aggregated sample included cohorts that were included in previous work, so our findings may mirror already published studies that compared (recreational and dependent) cannabis users to controls^17,26,27^. However, we were the first to concurrently examine the role of cannabis dependence status and sex differences on specific ROIs chosen based on their relevance for theories of addiction and their consistent alterations in regular cannabis users; also, studies that were published using samples from our aggregated sample were not used to compare our findings to already published work.

Last, we could not account for additional variables that may affect neuroanatomy in male and female cannabis users, including sex hormones^51,69^, cannabis use history and dependence severity^22^, craving^70^ and withdrawal^57^ motives to use cannabis (e.g., coping with stress, habits)^71,72^; cannabinoid compounds such as THC and cannabidiol (CBD), which might exacerbate or mitigate brain alterations^23^; stress level and psychiatric symptoms (e.g., anxiety, depression)^73,74^ and history of trauma^75–77^. This data was not available from this aggregated dataset and may reflect the status of the research to date, whereby distinct studies use heterogeneous measures of drug use, cognitive and psychological function. This situation may warrant the development of an expert-driven consensus on a minimum set of measures to map the brain, mental health and cognitive correlates of cannabis use. Such a consensus would be instrumental to help integrate research study findings and to advance the current understanding of the pathophysiology of cannabis use in men and women.

## Conclusions

In conclusion, we found that cannabis users compared to controls had smaller volumes in selected ROIs (i.e., cerebellar white matter and right lateral OFC). Smaller ROI volumes were predicted by female sex and presence of cannabis dependence. These results point to a role of cannabis dependence and female sex as drivers of subtle and regionally localized volumetric differences in cannabis users.

As cannabis becomes increasingly accessible to both men and women, more work is necessary to map the mechanisms underlying sex differences in trajectories in and out of cannabis dependence and related psychosocial problems. This, in turn, will help inform future research on sex-specific pharmacological and behavioural interventions for male and females regular and dependent cannabis users.

## Supplementary information

Supplement_Rossetti et al.

## Data Availability

The code of the statistical analysis and the datasets generated during and/or analysed during the current study are available from the corresponding author on reasonable request.

## References

[1] World Health Organization. *Global status report on alcohol and health, 2019*. World Health Organization 2019.

[2] Khan SS (2013). Gender differences in cannabis use disorders: results from the national epidemiologic survey of alcohol and related conditions. Drug Alcohol Depend..

[3] Ehlers CL (2010). Cannabis dependence in the San Francisco Family Study: age of onset of use, DSM-IV symptoms, withdrawal, and heritability. Addict. Behav..

[4] Hernandez-Avila CA, Rounsaville BJ, Kranzler HR (2004). Opioid-, cannabis-and alcohol-dependent women show more rapid progression to substance abuse treatment. Drug Alcohol Depend..

[5] Antinori, S. & Fattore, L. in *Endocannabinoids and Lipid Mediators in Brain Functions* (eds Melis, M.) Ch. 2 (Springer, Cham., 2017).

[6] Crane NA, Schuster RM, Fusar-Poli P, Gonzalez R (2013). Effects of cannabis on neurocognitive functioning: recent advances, neurodevelopmental influences, and sex differences. Neuropsychol. Rev..

[7] Everitt BJ, Robbins TW (2005). Neural systems of reinforcement for drug addiction: from actions to habits to compulsion. Nat. Neurosci..

[8] Glass M, Faull RLM, Dragunow M (1997). Cannabinoid receptors in the human brain: a detailed anatomical and quantitative autoradiographic study in the fetal, neonatal and adult human brain. Neuroscience.

[9] Lorenzetti V, Chye Y, Silva P, Solowij N, Roberts CA (2019). Does regular cannabis use affect neuroanatomy? An updated systematic review and meta-analysis of structural neuroimaging studies. Eur. Arch. Psychiatry Clin. Neurosci..

[10] Blithikioti C (2019). Cerebellar alterations in cannabis users: A systematic review. Addict. Biol..

[11] Lorenzetti V, Solowij N, Yücel M (2016). The role of cannabinoids in neuroanatomic alterations in cannabis users. Biol. Psychiatry.

[12] Mackey S (2019). Mega-analysis of gray matter volume in substance dependence: general and substance-specific regional effects. Am. J. Psychiatry.

[13] Koob GF, Volkow ND (2016). Neurobiology of addiction: a neurocircuitry analysis. Lancet Psychiatry.

[14] Ketcherside A, Baine J, Filbey F (2016). Sex effects of marijuana on brain structure and function. Curr. Curr. Addict. Rep..

[15] McQueeny T (2011). Gender effects on amygdala morphometry in adolescent marijuana users. Behav. Brain Res..

[16] Medina KL (2009). Prefrontal cortex morphometry in abstinent adolescent marijuana users: subtle gender effects. Addict. Biol..

[17] Chye Y (2017). Orbitofrontal and caudate volumes in cannabis users: a multi-site mega-analysis comparing dependent versus non-dependent users. Psychopharmacology.

[18] Price JS (2015). Effects of marijuana use on prefrontal and parietal volumes and cognition in emerging adults. Psychopharmacology.

[19] Medina KL, Nagel BJ, Tapert SF (2010). Abnormal cerebellar morphometry in abstinent adolescent marijuana users. Psychiatry Res..

[20] Gilman JM (2014). Cannabis use is quantitatively associated with nucleus accumbens and amygdala abnormalities in young adult recreational users. J. Neurosci..

[21] Lind KE (2017). Sex disparities in substance abuse research: evaluating 23 years of structural neuroimaging studies. Drug Alcohol Depend..

[22] Lorenzetti V (2020). Neuroanatomical alterations in people with high and low cannabis dependence. Aust. N. Z. J. Psychiatry.

[23] Yücel M (2016). Hippocampal harms, protection and recovery following regular cannabis use. Transl. Psychiatry.

[24] Battistella G (2014). Long-term effects of cannabis on brain structure. Neuropsychopharmacology.

[25] Hill SY, Sharma V, Jones BL (2016). Lifetime use of cannabis from longitudinal assessments, cannabinoid receptor (CNR1) variation, and reduced volume of the right anterior cingulate. Psychiatry Res.

[26] Solowij N (2011). Cerebellar white-matter changes in cannabis users with and without schizophrenia. Psychol. Med..

[27] Cousijn J (2012). Grey matter alterations associated with cannabis use: Results of a VBM study in heavy cannabis users and healthy controls. NeuroImage.

[28] Moreno-Alcázar A (2018). Larger grey matter volume in the basal ganglia of heavy cannabis users detected by voxel-based morphometry and subcortical volumetric analysis. Front. Psychiatry.

[29] Katona I. in *Behavioral neurobiology of the endocannabinoid system. current topics in behavioral neurosciences* Vol. 1 (eds Kendall, D. & Alexander, S.) Ch. 3 (Springer, Berlin, Heidelberg, 2009) 10.1007/978-3-540-88955-7_1 21104378

[30] Koob GF, Volkow ND (2010). Neurocircuitry of addiction. Neuropsychopharmacology.

[31] Miquel M (2016). Have we been ignoring the elephant in the room? Seven arguments for considering the cerebellum as part of addiction circuitry. Neurosci. Biobehav. Rev..

[32] Schacht JP, Hutchison KE, Filbey FM (2012). Associations between cannabinoid receptor-1 (CNR1) variation and hippocampus and amygdala volumes in heavy cannabis users. Neuropsychopharmacology.

[33] Batalla A (2014). Modulation of brain structure by catechol-O-methyltransferase Val(158) Met polymorphism in chronic cannabis users. Addict. Biol..

[34] Yücel M (2008). Regional brain abnormalities associated with heavy long-term cannabis use. Arch. Gen. Psychiatry.

[35] Chye Y (2017). Cannabis-related hippocampal volumetric abnormalities specific to subregions in dependent users. Psychopharmacology.

[36] Lecrubier Y (1997). The Mini International Neuropsychiatric Interview (MINI). A short diagnostic structured interview: reliability and validity according to the CIDI. Eur. Psychiatry.

[37] Gossop M (1995). The Severity of Dependence Scale (SDS): psychometric properties of the SDS in English and Australian samples of heroin, cocaine and amphetamine users. Addiction.

[38] Dale AM, Fischl B, Sereno MI (1999). Cortical surface-based analysis: I. Segmentation and surface reconstruction. Neuroimage.

[39] Desikan RS (2006). An automated labeling system for subdividing the human cerebral cortex on MRI scans into gyral based regions of interest. Neuroimage.

[40] Ségonne F (2004). A hybrid approach to the skull stripping problem in MRI. Neuroimage.

[41] Aarts E, Verhage M, Veenvliet JV, Dolan CV, Van Der Sluis S (2014). A solution to dependency: using multilevel analysis to accommodate nested data. Nat. Neurosci..

[42] Benjamini, Y. & Yekutieli, D. The control of the false discovery rate in multiple testing under dependency. *Annals Stat.* **29**, 1165-1188 (2001).

[43] Moulton EA, Elman I, Becerra LR, Goldstein RZ, Borsook D (2014). The cerebellum and addiction: insights gained from neuroimaging research. Addict. Biol..

[44] Goldstein RZ, Volkow ND (2011). Dysfunction of the prefrontal cortex in addiction: neuroimaging findings and clinical implications. Nat. Rev. Neurosci..

[45] Koob, G. F. in *Handb. Clin. Neurol*. 3rd edn, Vol. 125 (eds Sullivan, E. V. & Pfefferbaum, A.) Ch.3 (Elsevier B. V., 2014). 10.1016/B978-0-444-62619-6.09984-5 25307604

[46] Hirvonen J (2012). Reversible and regionally selective downregulation of brain cannabinoid CB 1 receptors in chronic daily cannabis smokers. Mol. Psychiatry.

[47] Kanaan RA (2012). Gender differences in white matter microstructure. PloS One.

[48] Ritchie SJ (2018). Sex differences in the adult human brain: evidence from 5216 UK biobank participants. Cereb. Cortex.

[49] Ruigrok ANV (2014). A meta-analysis of sex differences in human brain structure. Neurosci. Biobehav. Rev..

[50] Jacobus J (2013). White matter integrity, substance use, and risk taking in adolescence. Psychol. Addict. Behav..

[51] Farquhar CE (2019). Sex, THC, and hormones: Effects on density and sensitivity of CB1 cannabinoid receptors in rats. Drug Alcohol Depend..

[52] Fattore L, Fratta W (2010). How important are sex differences in cannabinoid action?. Br. J. Pharm..

[53] Romero J (1997). Atypical location of cannabinoid receptors in white matter areas during rat brain development. Synapse.

[54] Molina-Holgado E (2002). Cannabinoids promote oligodendrocyte progenitor survival: involvement of cannabinoid receptors and phosphatidylinositol-3 kinase/Akt signaling. J. Neurosci..

[55] Monnet-Tschudi F (2008). Delta-9-tetrahydrocannabinol accumulation, metabolism and cell-type-specific adverse effects in aggregating brain cell cultures. Toxicol. Appl. Pharmacol..

[56] Cutando L (2013). Microglial activation underlies cerebellar deficits produced by repeated cannabis exposure. J. Clin. Invest..

[57] Herrmann ES, Weerts EM, Vandrey R (2015). Sex differences in cannabis withdrawal symptoms among treatment-seeking cannabis users. Exp. Clin. Psychopharmacol..

[58] Craft RM, Marusich JA, Wiley JL (2013). Sex differences in cannabinoid pharmacology: a reflection of differences in the endocannabinoid system?. Life Sci..

[59] Cheetham A (2012). Orbitofrontal volumes in early adolescence predict initiation of cannabis use: a 4-year longitudinal and prospective study. Biol. Psychiatry.

[60] Sweigert J (2019). A multimodal investigation of cerebellar integrity associated with high‐risk cannabis use. Addict. Biol..

[61] Moorman DE (2018). The role of the orbitofrontal cortex in alcohol use, abuse, and dependence. Prog. Neuropsychopharmacol. Biol. Psychiatry.

[62] Durazzo, T. C. & Meyerhoff, D. J. Changes of frontal cortical subregion volumes in alcohol dependent individuals during early abstinence: associations with treatment outcome. *Brain Imaging Behav*. **14**, 1588-1599 (2020). 10.1007/s11682-019-00089-5 PMC6908760 31197582

[63] Weiland BJ (2015). Daily marijuana use is not associated with brain morphometric measures in adolescents or adults. J. Neurosci..

[64] Wetherill, R. R. et al. Cannabis, cigarettes, and their co-occurring use: disentangling differences in gray matter volume. *Int. J. Neuropsychopharmacol*. **18**, 116-123 (2015). 10.1093/ijnp/pyv061 PMC4648161 26045474

[65] Jacobus J (2015). Cortical thickness in adolescent marijuana and alcohol users: A three-year prospective study from adolescence to young adulthood. Dev. Cogn. Neurosci..

[66] Filbey FM, Gohel S, Prashad S, Biswal BB (2018). Differential associations of combined vs. isolated cannabis and nicotine on brain resting state networks. Brain Struct. Funct..

[67] Thompson PM (2014). The ENIGMA consortium: large-scale collaborative analyses of neuroimaging and genetic data. Brain Imaging Behav..

[68] van Erp TG (2016). Subcortical brain volume abnormalities in 2028 individuals with schizophrenia and 2540 healthy controls via the ENIGMA consortium. Mol. Psychiatry.

[69] Marusich JA, Craft RM, Lefever TW, Wiley JL (2015). The impact of gonadal hormones on cannabinoid dependence. Exp. Clin. Psychopharmacol..

[70] Wetherill RR, Jagannathan K, Hager N, Childress AR, Franklin TR (2015). Sex differences in associations between cannabis craving and neural responses to cannabis cues: Implications for treatment. Exp. Clin. Psychopharmacol..

[71] Köpetz CE, Lejuez CW, Wiers RW, Kruglanski AW (2013). Motivation and Self-Regulation in Addiction: A Call for Convergence. Perspect. Psychol. Sci..

[72] Sjoerds Z, Luigjes J, van den Brink W, Denys D, Yücel M (2014). The role of habits and motivation in human drug addiction: a reflection. Front. Psychiatry.

[73] Foster KT, Li N, McClure EA, Sonne SC, Gray KM (2016). Gender Differences in Internalizing Symptoms and Suicide Risk Among Men and Women Seeking Treatment for Cannabis Use Disorder from Late Adolescence to Middle Adulthood. J. Subst. Abus. Treat..

[74] Halladay JE, Boyle MH, Munn C, Jack SM, Georgiades K (2018). Sex differences in the association between cannabis use and suicidal ideation and attempts, depression, and psychological distress among Canadians. Can. J. Psychiatry.

[75] Everaerd D (2016). Childhood abuse and deprivation are associated with distinct sex-dependent differences in brain morphology. Neuropsychopharmacology.

[76] Chao T, Radoncic V, Hien D, Bedi G, Haney M (2018). Stress responding in cannabis smokers as a function of trauma exposure, sex, and relapse in the human laboratory. Drug Alcohol Depend..

[77] Elton A (2014). Childhood maltreatment is associated with a sex‐dependent functional reorganization of a brain inhibitory control network. Hum. Brain Mapp.

