# Supplementary material for: Sex and dependence related neuroanatomical differences in regular cannabis users: findings from the ENIGMA Addiction Working Group
Source: Transl Psychiatry. 2021 May 6;11:272. doi: 10.1038/s41398-021-01382-y (PMC8102553; doi:10.1038/s41398-021-01382-y)
Supplement: Supplementary file 1 — Supplement_Rossetti et al. [file 41398_2021_1382_MOESM1_ESM.pdf]

Sex and dependence related neuroanatomical differences in regular cannabis users: findings from the ENIGMA Addiction Working Group

Maria Gloria Rossetti, PhD,[1,2] Scott Mackey, PhD,[3] Praveetha Patalay, PhD,[4] Nicholas B. Allen PhD,[5] Albert Batalla, MD, PhD,[6] Marcella Bellani, MD, PhD,[1] Yann Chye, PhD,[7] Patricia Conrod, PhD,[8] Janna Cousijn, PhD,[9] Hugh Garavan, PhD,[3] Anna E. Goudriaan, PhD,[10] Robert Hester, PhD,[11] Rocio Martin-Santos, MD,[12] Nadia Solowij, PhD,[13] Chao Suo, PhD,[7] Paul M. Thompson, PhD,[14] Murat Yücel, PhD,[7] Paolo Brambilla, MD, PhD,[2,15] Valentina Lorenzetti, PhD[16]

[1] *Department of Neurosciences, Biomedicine and Movement Sciences, Section of Psychiatry, University of Verona, Verona, Italy*

[2] *Department of Neurosciences and Mental Health, Foundation IRCCS Ca'Granda Ospedale Maggiore Policlinico, University of Milan, Milan, Italy*

[3] *Department of Psychiatry, University of Vermont, Burlington, VT, USA*

[4] *Centre for Longitudinal Studies and MRC Unit for Lifelong Health and Ageing, IOE and Population Health Sciences, UCL, United Kingdom*

[5] *Department of Psychology, University of Oregon, Eugene, USA*

[6] *Department of Psychiatry, UMC Utrecht Brain Center, University Medical Centre Utrecht, Utrecht, The Netherlands*

[7] *Turner Institute for Brain and Mental Health, School of Psychological Sciences, Monash University, Clayton, VIC, Australia*

[8] *Department of Psychiatry, Université de Montreal, CHU Ste Justine Hospital, Montreal, QC, Canada*

[9] *Department of Psychology, University of Amsterdam, Amsterdam, the Netherlands*

[10] *Department of Psychiatry, Amsterdam Institute for Addiction Research, University of Amsterdam, Amsterdam, Netherlands*

[11] *School of Psychological Sciences, University of Melbourne, Melbourne, Australia*

[12] *Department of Neurosciences and Behavior, Ribeirão Preto Medical School, University of São Paulo, Av. Bandeirantes, Ribeirão Preto, São Paulo, Brazil*

[13] *School of Psychology and Illawarra Health and Medical Research Institute, University of Wollongong, Wollongong, Australia*

[14] *Departments of Neurology, Radiology, Engineering, & Psychiatry, Imaging Genetics Center, Mark & Mary Stevens Institute for Neuroimaging & Informatics, Keck School of Medicine, University of Southern California, Marina del Rey, CA, USA*

[15] *Department of Pathophysiology and Transplantation, University of Milan, Milan, Italy*

[16] *Neuroscience of Addiction & Mental Health Program, Healthy Brain and Mind Research Centre, School of Behavioural & Health Sciences, Faculty of Health Sciences, Australian Catholic University, Melbourne, VIC, Australia*

Assessment instrument by imaging site

Supplementary Table S1 shows differences in behavioral, mental health, substance use and MRI assessment tools by imaging site. Cannabis use was measured as grams, joints and cones or as occasions of use. Grams and joints were converted to 'cones' as cones is a standardized unit for comparisons (e.g., 1g = 12 cones, 1 paper joint = 3 cones; see guidelines provided at https://cannabissupport.com.au/media/1593/timeline-followback.pdf). Alcohol and tobacco use was quantified as standard drinks and number of cigarettes respectively, as well as days/occasions of use. For statistical analyses we adopted the measures that were more consistently used across sites including, age of onset of use and monthly cones for cannabis; monthly standard drinks for alcohol and monthly cigarettes for tobacco (measures expressed in days/weeks were previously converted to months).

The Intelligent Quotient (IQ) was estimated using the Dutch version of the National Adult Reading Test (DART) (1) (site 1); the National Adult Reading Test (NART) (2) (site 3) and The Wechsler Abbreviated Scale of Intelligence (WASI) (3) (site 4). The vocabulary subscale score of the Wechsler Adult Intelligence Scale, 3rd ed. (WAIS-III) (4) from the site 2 was converted to an estimated full scale IQ by standardizing to the average IQ of cannabis users and controls respectively of the remaining sites (5).

Supplementary Table S1. Assessment instruments by imaging site

	Site 1 Amsterdam [6] *(N =73)*	***Site 2 Barcelona*** [7] *(N=59)*	***Site 3 Wollongong*** [8] *(N=34)*	***Site 4 Melbourne*** [9] *(N=101)*
**Inclusion criteria**				
Cannabis group	≥ 10 days/mo, ≥ 240 days over last 2 yrs	First use age < 16, 14-28 joints/week over last 2 yrs	≥ 3-4 times/wk	≥ 2 days/mo for at least 2 yrs
Control group	< 50 joints//lifetime, did not use in last mo	< 15 lifetime experiences with cannabis	No lifetime history of regular cannabis use, did not use in last yr	</= 10 times/lifetime, did not use in last yr
**Exclusion criteria, all participants**				
IQ	< 80	< 90		< 70
Axis I psychiatric disorders	MINI	PRISM	SCID	SCID
Illicit substance use other than cannabis	> 100 times/lifetime	> 5 times/lifetime	√	> 50 times/past 10 yrs
Positive toxicology (urine) test	√	√	√	√
Female		√		
MR contraindication	√	√	√	√
Use of psychoactive medication	√	√	√	√
Left-handed		√	√	
**Assessment tools**				
IQ	DART	WAIS-III vocabulary subscore	NART	WASI
**Cannabis use**				
Onset of use, yrs	√	√	√	√
Frequency	Days/wk		Days/mo	Days/mo
Dosage	Grams/wk,	Joints/day	Cones/mo	Cones/mo
Dependence	MINI – score >/= 3 as dependent	SDS – score >/= 4 as dependent	SCID	SDS – score >/= 4 as dependent
**Alcohol use**				
Frequency				
Dosage	StDr/mo	StDr/wk	StDr/wk	StDr/mo
**Tobacco use**				
Frequency				
Dosage	Cig/day	Cig/day	Cig/day	Cig/mo

Wk = week, mo = month, yrs = years, occ= occasions, StDr = standard drinks, cig = cigarettes, MINI = Mini Neuropsychiatry International Interview (10), PRISM = Psychiatric Research Interview for Substance and Mental Disorders (http://www.columbia.edu/~dsh2/prism/) (11), SCID = Structured Clinical Interview for DSM Disorders (12), DART = Dutch version of the National Adult Reading Test (1), WAIS-III = Wechsler Adult Intelligence Scale – Third Edition (4), NART = National Adult Reading Test (2), WASI = Wechsler Abbreviated Scale of Intelligence (3), SDS = Severity of Dependence Scale (13)

Supplementary Table S2. Scanners parameters by imaging site

	**Site 1 Amsterdam** (N =73)	**Site2 Barcelona** (N=59)	**Site 3 Wollongong** (N=34)	**Site 4 Melbourne** (N=101)
Manufacturer and Model	Phillips Intera	GE Signa Ecite	Phillips Intera	Siemens Trio
Field Strength (Tesla, T)	3	1.5	3	3
Number of Channels	8	8	8	32
Repetition Time, TR (ms)	9600	11.8	6.4	1900
Echo Time, TE (ms)	4.6	4.2	2.9	2.15
Sequence	Turbo field echo	3D inversion recovery prepared fast spoiled gradient	3D spoiled gradient-recalled echo	MPRAGE

Supplementary Table S3. Demographic and substance use characteristics of participants from the three-site subsample with data for cannabis dependence status (Mean (SD))

	HC		Recreational CB		Dependent CB		*Group*						*Sex*		*Group-by-Sex*		Site§
							(dependent CB vs recreational CB)		*(dependent CB vs HC)*		*(recreational CB vs HC)*		*(Males vs Females)*				
	Males	*Females*	*Males*	*Females*	*Males*	*Females*	*β* (95% CI)	*p*	*β* (95% CI)	*p*	*β* (95% CI)	*p*	*β* (95% CI)	*p*	*β* (95% CI)	*p*	Var
Sex, N	65	33	29	20	42	17	-				-		-	-	-	-	-
Age, yrs	24.02 (7.20)	25.50 (9.06)	23.74 (8.62)	31.75 (11.20)	24.91 (8.07)	26.46 (9.12)	-3.32 (-6.20, 0.46)	.023*	1.08 (-3.62, 1.45)	.401	2.24 (-0.27, 4.75)	.080	-0.11 (-2.45, 2.23)	.924	1.24	.624	.30
IQ	108.18 (12.57)	106.21 (11.70)	99.66 (14.02)	104.15 (9.32)	99.83 (12.00)	98.53 (11.08)	-2.66 (-7.41, -2.08)	.271	-8.42 (-12.60, 4.23)	<.001***	-5.75 (-9.91, 1.59)	<.007**	1.71 (-2.09, 5.52)	.377	-0.27 (-8.51, 7.97)	.948	.06
Alcohol, StDr/mo	19.72 (22.98)	17.60 (23.10)	28.21 (37.38)	17.05 (23.06)	32.93 (40.01)	20.82 (14.81)	4.30 (-7.10, 15.70)	.460	7.28 (-2.77, 17.32)	.156	2.98 (-7.01, 12.96)	.559	13.04 (3.93, 22.16)	.005	10.89 (-8.911, 30.69)	.281	.04
Tobacco, Cig/mo	25.50 (94.69)	51.09 (110.03)	207.65 (236.98)	289.97 (266.93)	220.98 (207.70)	242.00 (211.00)	-17.71 (-52.82, 88.23)	.623	192.19 (-130.32, 254.06)	<.001***	209.90 (147.75, 272.04)	<.001***	-40.08 (-93.65, 13.50)	.143	5.34 (-118.04, 128.73)	.932	.00
Cannabis Onset of use, yrs	-	-	14.97 (1.88)	15.70 (2.47)	15.19 (1.71)	14.41 (1.66)	-0.68 (-1.82, 0.46)	.245	-	-	-	-	0.73 (-0.26, 1.71)	.149	0.07 (-2.19, 2.34)	.949	.05
Dosage, cones/mo	-	-	252.66 (191.88)	200.90 (228.56)	334.76 (317.09)	318.12 (191.10)	84.44 (13.17, 155.16)	.019*	-	-	-	-	52.22 (-4.71, 109.17)	.072	29.84 (-92.87, 152.56)	.634	.07
ICV, 10$^{\wedge 6}$	1.60 (0.15)	1.40 (0.17)	1.54 (0.14)	1.34 (0.19)	1.57 (0.15)	1.48 (0.14)	0.05 (-0.00, 0.11)	.059	-0.01 (-0.06, 0.04)	.763	-0.06 (-0.11, 0.01)	.014	0.17 (-0.22, 0.13)	<.001***	-0.09 (-0.18, 0.01)	.079	.19

β = beta; CB = cannabis users; CI = confidence interval; Cig = cigarettes; Cones = standardized measure of cannabis dosage, see https://cannabissupport.com.au/media/1593/timeline-followback.pdf; HC = controls; ICV = Intracranial volume; IQ = Intelligence quotient; mo = monthly; StDr = Standard drinks; Var = Variation, yrs = years. § Site level variation based on intraclass correlation (ICC). Differences between groups for sex distribution were measured with chi^2 test (χ^2=1.73, p = 0.42).

* *p(unc)* <.05, ***p(unc)* <.01, *** *p(unc)* < .001.

References

1. Schmand B, Bakker D, Saan R, Louman J. The Dutch Reading Test for Adults: a measure of premorbid intelligence level. *Tijdschrift voor gerontologie en geriatrie* 1991; 22(1): 15-19.

2. Nelson HE, Willison J. *National Adult Reading Test (NART)*. Nfer-Nelson Windsor 1991.

3. Wechsler D. Manual for the Wechsler Abbreviated Scale of Intelligence. 1999.

4. Wechsler, D. (1997). Wechsler Memory Scale (3rd edn.). San Antonio, TX: Psychological Corporation.

5. Chye Y, Solowij N, Suo C, Batalla A, Cousijn J, Goudriaan AE *et al.* Orbitofrontal and caudate volumes in cannabis users: a multi-site mega-analysis comparing dependent versus non-dependent users. *Psychopharmacology* 2017; 234(13): 1985-1995.

6. Cousijn J, Goudriaan AE, Ridderinkhof KR, van den Brink W, Veltman DJ, Wiers RW. Approach-bias predicts development of cannabis problem severity in heavy cannabis users: results from a prospective FMRI study. *PloS one* 2012; 7(9): e42394.

7. Batalla A, Soriano-Mas C, Lopez-Sola M, Torrens M, Crippa JA, Bhattacharyya S *et al.* Modulation of brain structure by catechol-O-methyltransferase Val(158) Met polymorphism in chronic cannabis users. *Addict Biol* 2013.

8. Yücel M, Solowij N, Respondek C, Whittle S, Fornito A, Pantelis C *et al.* Regional brain abnormalities associated with heavy long-term cannabis use. *Archives of General Psychiatry* 2008; 65(6): 1 - 8.

9. Chye Y, Suo C, Yücel M, Den Ouden L, Solowij N, Lorenzetti V. Cannabis-related hippocampal volumetric abnormalities specific to subregions in dependent users. *Psychopharmacology* 2017: 1-9.

10. Sheehan DV, Lecrubier Y, Sheehan KH, Amorim P, Janavs J, Weiller E *et al.* The Mini-International Neuropsychiatric Interview (MINI): the development and validation of a structured diagnostic psychiatric interview for DSM-IV and ICD-10. *The Journal of Clinical Psychiatry* 1998.

11. Torrens M, Serrano D, Astals M, Pérez-Domínguez G, Martín-Santos R. Diagnosing comorbid psychiatric disorders in substance abusers: validity of the Spanish versions of the Psychiatric Research Interview for Substance and Mental Disorders and the Structured Clinical Interview for DSM-IV. *American Journal of Psychiatry* 2004; 161(7): 1231-1237.

12. First MB. Structured clinical interview for DSM-IV axis I disorders. *Biometrics Research Department* 1997.

13. Gossop M, Darke S, Griffiths P, Hando J, Powis B, Hall W *et al.* The Severity of Dependence Scale (SDS): psychometric properties of the SDS in English and Australian samples of heroin, cocaine and amphetamine users. *Addiction* 1995; 90(5): 607-614.
